# Differences in Total Daily Energy Expenditure Across Field Sports: A Narrative Review

**DOI:** 10.3390/jfmk10040474

**Published:** 2025-12-09

**Authors:** Brenen Skalitzky, Jennifer B. Fields, Margaret T. Jones, Chad M. Kerksick, Andrew R. Jagim

**Affiliations:** 1 School of Medicine and Public Health, University of Wisconsin–Madison, Madison, WI 53706, USA; bskalitzky@wisc.edu; 2 Patriot Performance Laboratory, Frank Pettrone Center for Sports Performance, George Mason University, Fairfax, VA 22030, USA; jennifer.fields@uconn.edu (J.B.F.); mjones15@gmu.edu (M.T.J.); 3 Department of Nutritional Sciences, University of Connecticut, Storrs, CT 06269, USA; 4 Sport, Recreation, and Tourism Management, George Mason University, Fairfax, VA 22030, USA; 5 Exercise and Performance Nutrition Laboratory, Lindenwood University, St. Charles, MO 63301, USA; ckerksick@lindenwood.edu; 6 Sports Medicine, Mayo Clinic Health System, La Crosse, WI 54601, USA; 7 Department of Exercise and Sport Science, University of Wisconsin–La Crosse, La Crosse, WI 54601, USA

**Keywords:** total daily energy expenditure, doubly labeled water, physical activity level, athlete nutrition, sport-specific energy needs, energy availability, sports physiology

## Abstract

**Background**: Differences in total daily energy expenditure (TDEE) across sports, sex, and skill level support the need for sport- and athlete-specific energy intake recommendations. The purpose of the current review was to examine TDEE and related markers of energy expenditure across field-based team sports. A secondary aim was to evaluate physical activity levels (PAL), calculated as TDEE divided by resting metabolic rate (RMR), and their utility in estimating energy needs within team sports. **Methods**: The review was limited to studies that included the field-based team sports of rugby or soccer and reported energy expenditure data using doubly labeled water (DLW). A literature review identified 11 studies meeting criteria. Weighted means (Xw) and standard deviations (SDw) were calculated for each variable when pooled across each sport category. **Results**: Rugby (4417 ± 654 kcal·d^−1^) had a higher average TDEE than soccer (3157 ± 331 kcal/day; *p* < 0.001). When normalized to body mass, rTDEE was similar between sports (rugby: 49.5 ± 1.3 kcal·kg^−1^·day^−1^; soccer: 49.3 ± 1.8 kcal·kg^−1^·day^−1^; *p* = 0.967). PAL values were significantly higher in rugby (2.2 ± 0.4) compared to soccer (1.7 ± 0.2; *p* = 0.004). RMR was also greater in rugby (2136 ± 322 kcal·d^−1^) compared to soccer (1835 ± 208 kcal·d^−1^; *p* = 0.04). **Conclusions**: Rugby athletes exhibited higher TDEE values than soccer athletes, reflecting greater absolute energy demands. However, similar relative TDEE values suggest that differences in body size and composition likely contribute to the observed differences in absolute expenditure. These findings underscore the importance of individualized nutrition strategies within team sports and highlight PAL as a useful metric to contextualize energy requirements.

## 1. Introduction

Optimizing energy intake to match energy expenditure is a foundational principle of sports nutrition [1,2,3]. Athletes require sufficient energy not only to support sport-specific training and competition demands, but also to maintain health, promote recovery, and reduce the likelihood of training in a low energy availability (LEA) state [3]. Total daily energy expenditure (TDEE) is a critical variable in the development of personalized nutrition strategies for athletes, as it reflects the energy required to support basal physiological functions in addition to daily physical activity [4,5]. Differences in energy expenditure across sports, sex, and training demands necessitate sport-specific and athlete-specific assessments of TDEE to guide energy intake recommendations [2]. Inadequate energy availability resulting from misalignment between TDEE and intake can impair health and performance [3]. Therefore, the identification of TDEE values across sports is a key step in the development of evidence-based, individualized nutrition plans for athletes.

Total daily energy expenditure consists of three primary components, including resting metabolic rate, thermic effect of food, and physical activity energy expenditure [5]. Resting metabolic rate (RMR) includes the energy required to maintain essential physiological functions at rest, typically comprising 60–75% of TDEE in sedentary individuals [5,6]. The thermic effect of food (TEF) includes the energy cost of digesting, absorbing, and metabolizing food, generally equating to ~10% of energy intake [7]. Lastly, physical activity energy expenditure (PAEE) reflects energy expended during exercise and non-exercise activity thermogenesis (NEAT). In athletic populations, PAEE contributes the most variable component of TDEE, influenced by sport type, training volume, sex, body mass, and competition season [4,5]. Recognizing and quantifying these factors are essential to avoid the consequences of energy imbalance, including impaired metabolic, hormonal, immune, and psychological function, as well as diminished sport performance [3].

The gold-standard method for assessing TDEE in free-living ambulatory settings is doubly labeled water (DLW) [8,9], a stable isotope technique that measures CO_2_ production over time, allowing for accurate estimation of energy expenditure. While highly valid, DLW is expensive and logistically complex, making it impractical for widespread use in field settings. Other methods used to estimate TDEE in athletes include indirect calorimetry (for RMR), accelerometry and heart rate monitoring (often including wearable metabolic devices), activity logs and metabolic equivalents (METs), and other prediction equations based on body mass and training hours [10,11,12,13,14]. While each method has its limitations, combining wearable monitoring with athlete-reported training logs can provide reasonably accurate TDEE estimates when DLW is not feasible [10,11,12,13,14].

Individual energy expenditure values and metabolic requirements are largely influenced by body size, specifically fat-free mass (FFM) [15,16,17]. A physical activity level (PAL) serves as a key metric used to contextualize a person’s daily energy expenditure in relation to their own metabolic requirements [18,19]. Physical activity levels are defined as: PAL = TDEE/RMR and reflects the overall activity intensity of a given day and is used to estimate TDEE from known or measured RMR values [18,19]. General population PAL values range from ~1.2 (sedentary) to 2.5 (very active). In athletes, PALs can reach values above 3.0, depending upon training volume and sport demands [4]. Using PAL values enables practitioners to estimate TDEE from RMR, which can be measured using indirect calorimetry or estimated using validated prediction equations [20].

Existing research on TDEE in athletes has traditionally focused on endurance athletes [4] and single-sport investigations [21,22,23]. Much of the foundational literature, including systematic reviews such as Heydenreich et al. [4], centers on endurance disciplines and characterizes their seasonal fluctuations in TDEE, energy intake, and energy availability. While those studies provide important insights into high-volume endurance training demands, they offer limited applicability to field-based team sports, whose intermittent, collision-based, and multi-directional movement patterns create fundamentally different metabolic demands. Other available studies [21,22,24], particularly those within rugby or soccer, tend to be isolated investigations using DLW but within a single team, sex, or age group. While valued, these studies lack the cross-sport comparative perspective necessary to contextualize energy needs across team sport populations.

In contrast, this narrative review compares TDEE values across two of the most widely played field-based team sports, rugby and soccer, using only DLW-derived data. By restricting inclusion criteria exclusively to DLW studies, the present review synthesizes the highest-quality evidence available, whereas many existing reviews blend methodologies (accelerometry, prediction equations, heart-rate models), resulting in greater variability and reduced precision. This methodological specificity ensures that comparisons between sports are physiologically meaningful and not confounded by measurement error inherent in other estimation techniques.

Previous work has indicated that athletes exhibit high TDEE values during training, predisposing them to energy deficiencies when energy intake is not adjusted to support the high energy expenditures [4,22,25,26,27,28,29]. There is a critical need to identify sport-specific energy requirements to provide precise recommendations for energy intake requirements for the optimization of athlete performance and health. This requires a review of the literature, focusing on gold-standard practices of quantifying TDEE. Therefore, the purpose of the current review was to examine TDEE and related markers of energy expenditure across field-based team sports of rugby and soccer. A secondary aim was to evaluate PALs and their utility in estimating energy needs within said sports. We hypothesized that soccer athletes would exhibit a higher TDEE yet lower rTDEE compared to rugby athletes.

## 2. Materials and Methods

The authors conducted a systematic search and narrative synthesis to identify previously published articles that quantified TDEE in athletic populations. Studies were limited to those that used DLW as the primary method of determining TDEE. Additional inclusion criteria included:

• Athletic populations competing in soccer or rugby.
• Studies including both male and female athletes.
• Athletes across all levels of competition were considered.
• Published in English.
• Published across any timeframe.

A comprehensive literature search was conducted using PubMed, Scopus, Web of Science, CINAHL, and EBSCOHOST. Published original research from any time period was included as long as it met the inclusions criteria listed above. The Medical Subject Headings search terms included keywords such as “energy expenditure,” “total daily energy expenditure,” “daily energy expenditure,” “calorie expenditure,” “energy balance,” “energy requirements,” “activity levels,” “calorie requirements,” “doubly labeled water,” “rugby OR soccer.” The literature review identified 11 studies meeting the inclusion criteria. Each study was reviewed for athlete demographics, performance level, and reported values for TDEE, relative TDEE, RMR, and PAL.

### Statistical Analysis

Weighted means (Xw) and standard deviations (SDw) were calculated for each variable when pooled across each sport category. Studies that included multiple time points across the phases of the competitive season were treated as independent data points. Independent samples *t*-tests were used to assess differences across sport categories (*p* < 0.05). Mean differences and 95% confidence intervals along with Cohen’s d effect sizes (ES) were also calculated to allow for a better interpretation of the magnitude of differences observed. Effect sizes were interpreted as follows: large (d > 0.8), moderate (d = 0.8–0.5), small (d = 0.49–0.20), and trivial (d < 0.2) [30]. All data were analyzed using the IBM SPSS Statistics for Windows (Version 26.0; IBM Corp., Armonk, NY, USA).

## 3. Results

A summary of all reported data, separated between rugby and soccer athletes, can be seen in Table 1 and Table 2.

**Figure 1:**
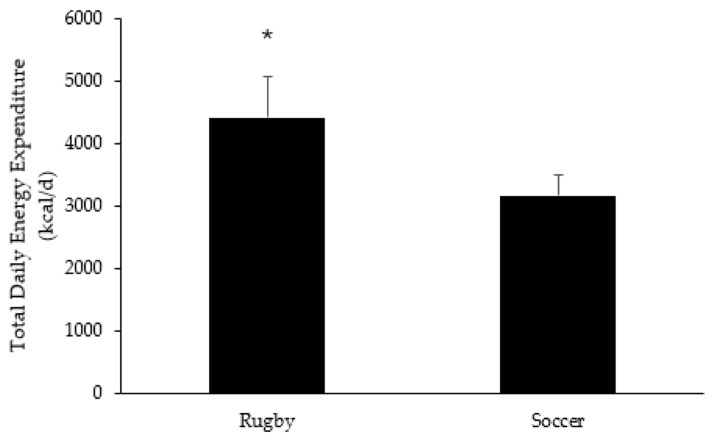
Total Daily Energy Expenditure by Sport Type. * Denotes significant differences between sport groups.

Individual statistics from the analyzed studies can be found in Table 2. Rugby (4417 ± 654 kcal·d^−1^) had a higher average TDEE than soccer (3157 ± 331 kcal·d^−1^; *p* < 0.001; ES = 2.4) as seen in Figure 1. Across all studies, rugby averaged 1258 ± 197 kcal·d^−1^ (95% confidence intervals: 849, 1666 kcal·d^−1^) higher TDEE compared to soccer. Total daily energy expenditure was then normalized to body mass (rTDEE). Using this approach, rTDEE was similar between sports (rugby: 49.5 ± 1.3 kcal·kg^−1^·day^−1^); soccer: 49.3 ± 1.8 kcal·kg^−1^·day^−1^); *p* = 0.967; ES = 0.13), with a mean difference of 0.62 kcal·kg^−1^·day^−1^ (95% CI: −11.3, 12.5 kcal·kg^−1^·day^−1^).

RMR was greater in rugby athletes (2136 ± 322 kcal·d^−1^) compared to soccer athletes (1835 ± 208 kcal·d^−1^; *p* = 0.04; ES = 1.1). Across all studies, rugby averaged 302 kcal·d^−1^ (95% confidence intervals: 67. 536 kcal·d^−1^) higher RMR compared to soccer. For PALs, values were significantly higher in rugby (2.2 ± 0.4) compared to soccer (1.7 ± 0.2; *p* = 0.004; ES = 1.6), which resulted in a mean difference of 0.42 (95% CI: 0.15, 0.68).

## 4. Discussion

The main focus of the current review was to examine TDEE and related markers of energy expenditure across two popular field-based team sports: rugby and soccer. The main findings from the current review indicate that rugby had a higher average TDEE compared to soccer (Figure 1). However, when normalized to body mass, rTDEE was similar between sports, which did not support our current hypothesis.

### 4.1. Bioenergetic Demands of Team Sports

Understanding sport-specific bioenergetic demands is crucial for effective conditioning, recovery, and nutrition planning. To some degree, each sport exhibits a distinct bioenergetic profile, which subsequently influences sport-specific energy expenditures and overall TDEE, along with specific macronutrient requirements. The primary sports included in the current review, rugby and soccer, vary significantly in structure, kinematic profiles, and gameplay, which likely influenced the observed differences in energy expenditure. Rugby Union is played on a 100 × 70-m pitch with 15 players per team and consists of two 40-min halves. Rugby players exhibit frequent collisions and tackles, anaerobic sprints, and prolonged aerobic effort, requiring both dynamic strength and endurance [36,37,38]. Elite Rugby Union, athletes cover 5550–6100 m, depending on position, with 300–400 m performed as high-intensity running [39]. Soccer is played on a pitch with similar dimensions, typically between 100–110 × 65–75 m, with 11 players per team playing two 45-min halves. Soccer players exhibit continuous aerobic movement with frequent high-speed bursts [40,41,42,43]. At elite levels, soccer athletes can travel ~10 km in a match, with nearly a quarter of that distance being high-intensity or sprinting. These differences in field dimensions, team composition, duration of play, and movement dynamics contribute to sport-specific variations in TDEE and bioenergetic demands. Contextual variables, including the level of play, variations in regulation game duration, as well as sport position responsibilities within the team contribute to nuanced inter-sport differences between individual players [41,44,45]. Due to the unique bioenergetic and physiological demands of each sport, differences in athlete body size and composition are frequently observed, as some body types are better suited for specific sports [46,47,48]. Therefore, while match demands and body size influence absolute measures of TDEE, relative TDEE is a more individualized indicator of an athlete’s energy requirements.

### 4.2. TDEE and PAL Values Across Sports

Energy demands in sports vary by sex, player position, level of competition, and match duration. For example, male soccer players exhibit TDEE values ranging from ~3000 to 3500 kcal·d^−1^. Whereas TDEE in male rugby athletes appear to be much higher at ~4400 kcal·d^−1^. These differences in TDEE are likely underpinned by differences in body size as when expressed as relative TDEE, values are nearly equal between soccer and rugby at 49 kcal·kg^−1^·day^−1^ (Table 2). Female soccer athletes tend to have lower TDEE values, ranging from 2500 to 3400 kcal·d^−1^. When expressed relative to body mass, TDEE values are closer to those of their male counterparts (Table 2). Although limited data exist relative to women’s rugby, results from the current review indicate similar TDEE values between women’s rugby and soccer with the findings from Wilson et al. [31], demonstrating an average TDEE value of 3229 ± 545 kcal·d^−1^ in international female rugby union players during an international tournament. Further research is needed to quantify seasonal trends in TDEE and to identify individual energy requirements for women’s rugby and soccer. Previous work has highlighted the high degree of variability when quantifying TDEE values in sport. In a cohort of elite junior basketball players, Silva et al. [49,50] measured TDEE values ranging from ~3600–4200 kcal·d^−1^ during training and competition phases.

Another key finding from the current review is the presentation of both absolute and relative TDEE comparisons. Prior research typically reports absolute energy expenditure without accounting for body mass differences, something that strongly influences metabolic rate and overall energy expenditure. By presenting both absolute TDEE and rTDEE (kcal·kg^−1^·day^−1^), this review clarifies that while rugby players exhibit significantly higher absolute TDEE values, relative TDEE is essentially identical between rugby and soccer. This finding fills an important gap by demonstrating that differences in body size, not differences in physiological effort, primarily drive the variation in absolute energy demands, an insight absent in earlier sport-specific papers. In the current review, when accounting for individuals’ resting metabolism, the resulting PAL values identified in rugby and soccer were similar, with values ranging between ~2.1 and 2.7.

In athletes, PAEE can account for 15–50% or more of TDEE, depending on training load. This variability in PAEE and TDEE highlights the importance of nutritional periodization strategies to ensure sufficient fueling practices during periods of intensive training and competition. However, investigations have consistently demonstrated that reported dietary (energy) intakes often fail to adequately match the high TDEE values in various athlete populations. This phenomenon is particularly evident in endurance athletes, as a previous systematic review indicated that male endurance athletes exhibit an energy deficit of −304 kcal·d^−1^, on average, during the preparation phase and ~2000 kcal·d^−1^ during the competition phase of their season [4]. Similar findings were reported in female endurance athletes, with a negative energy balance of −1145 kcal·d^−1^, observed during the preparation phase and the competition phase (−1252 kcal·d^−1^). These energy deficits equated to relative energy deficits of 6.6% of TDEE during the preparation phase and 18.9% during the competition phase in the male endurance athletes, and 29.0% of TDEE during the preparation phase, and 22.0% during the competition phase for the female endurance athletes. Less is known regarding the energy status of field-based athletes when using DLW for TDEE determination. From the current review, findings from Dasa et al. [33] documented a mean TDEE of 2918 ± 322 kcal·d^−1^ in female professional soccer players, yet mean energy intake was only 2274 ± 450 kcal·d^−1^, a deficit of ~22%. Amongst a cohort of male academy soccer players, Hannon et al. [34] reported a mean TDEE of 3586 ± 487 kcal·d^−1^, while consuming only 3180 ± 279 kcal·d^−1^, producing a negative balance of ~400 kcal·d^−1^. Similar gaps were identified in rugby athletes, where Costello et al. [32] observed professional young players expending ~3862–4384 kcal·d^−1^ but consuming only 3231–3357 kcal·d^−1^, again leaving a consistent daily deficit of ~500–700 kcal. These data illustrate that TDEE is influenced not only by sex and sport but also by training intensity and body size, subsequently resulting in varying degrees of energy availability status, based on dietary intake. Maintaining energy balance is critical for both short-term and long-term athlete health and performance [3]. When intake consistently falls short of TDEE, athletes may be in a state of negative energy balance and may experience low energy availability (LEA), defined as the energy remaining for physiological functions after accounting for exercise energy expenditure [3,51]. When contextualized within the Relative Energy Deficiency in Sport (RED-S) framework [3], these findings highlight a persistent risk for LEA among team sport athletes. Even relatively modest daily deficits (e.g., 300–700 kcal) can accumulate over weeks of training and competition, contributing to endocrine disturbances, reduced recovery capacity, and impaired performance [3]. Thus, across both field-based, endurance-based, and other team sports, the literature converges on a critical theme: high measured TDEE values are frequently unmatched by energy intake, underscoring the importance of regular monitoring, nutrition education, and individualized fueling strategies.

### 4.3. Using TDEE and PAL to Personalize Athlete Nutrition

The review also addresses a critical gap regarding physical activity levels (PAL) in team sports. PAL is often referenced in the general population’s literature, but limited synthesis exists on PAL values specifically in elite team sport athletes. By extracting and pooling sport-specific PAL values, this review provides practitioners with practical multipliers that can be applied to measured or predicted resting metabolic rate (RMR) to estimate TDEE when DLW is not feasible. This is a meaningful contribution, as many previously published studies report TDEE values but do not contextualize them relative to RMR or provide actionable PAL ranges for field application. Given the wide range of TDEE values across and within sports, when designing individualized nutrition planning, the direct measurement or estimation of RMR should be considered. Once this is established, the utilization of a corresponding PAL based on training load and information (e.g., Table 2) can allow for the multiplication of RMR × PAL to estimate TDEE and determine an athlete’s energy requirements. Lastly, there may be a need for an adjustment of energy intake to reflect current goals (e.g., maintenance, gain, or loss).

For example: A female soccer player with a measured RMR of 1400 kcal·d^−1^ and an estimated PAL of 2.0 (previously shown in female soccer athletes [33]) would have an estimated TDEE of 2800 kcal·d^−1^ as shown below:

(1) TDEE = 1400 × 2.0 = 2800 kcal·d^−1^

The athlete’s energy intake should be set near this value to maintain body mass or adjusted based on specific performance or weight management goals.

To apply TDEE and PAL in practice, practitioners can utilize validated RMR prediction equations when indirect calorimetry is unavailable. Secondly, it is important to assign sport-specific PALs (e.g., Table 2 or relevant sport science resources). It may also be important to reassess TDEE periodically across macrocycles (pre-season, in-season, off-season) and monitor body mass, performance, recovery, and any unique nutritional goals. Further, education of athletes and coaches on energy balance and the risks associated with LEA is warranted to promote prioritization of optimal nutrition strategies throughout the season.

### 4.4. Future Directions and Research Needs

This review identifies significant sex-specific and youth data gaps, particularly the scarcity of DLW-based TDEE values in female rugby athletes and broader underrepresentation of youth athletes outside academy-level soccer. By highlighting these gaps, the review not only synthesizes current knowledge but also maps clear directions for future research. Collectively, this review advances the literature by integrating gold-standard DLW data across multiple competition levels, sexes, and age groups within two major field sports, providing the first clear cross-sport comparison of TDEE, rTDEE, RMR, and PAL, and translating these findings into practical, individualized nutrition planning implications.

### 4.5. Limitations

Several limitations should be considered when interpreting the findings of this narrative review. First, because the analysis was restricted to studies employing the DLW method to quantify TDEE, this reduced the number of eligible studies. As a result, the data synthesized here represent only a subset of available research on rugby and soccer athletes, and the findings may not fully capture the breadth of energy expenditure patterns across all competition levels or geographic regions. Furthermore, DLW studies often involve small sample sizes due to methodological cost and complexity, which may reduce generalizability, particularly within subgroups such as female athletes, youth players, and position-specific roles.

Second, the review did not include other team sports beyond soccer and rugby. This decision was intentional to maintain methodological rigor and direct comparability; however, it limits the ability to extrapolate findings to other field-based sports such as American football, lacrosse, field hockey, or Australian rules football, where movement profiles, collision demands, and metabolic stressors differ substantially. Future work expanding DLW-based TDEE assessments across a broader range of team sports and both sexes would further clarify sport-specific energy requirements.

Third, heterogeneity in the included studies may influence the pooled results. Differences in competitive season (pre-season vs. in-season), training schedules, body composition (i.e., fat-free mass differences, body size, etc.) match frequency, environmental conditions, and nutritional behaviors may all contribute to variation in TDEE, RMR, and PAL. Some studies included tournament settings or atypical training loads, which may not reflect habitual energy expenditure across an entire season. Additionally, several studies did not report key variables such as body composition, positional roles, or daily training intensity, limiting the ability to identify precise contributors to inter-athlete variability.

Finally, the cross-sectional nature of most included studies restricts insight into temporal changes in TDEE across training cycles. Seasonal fluctuations in training load, match density, and recovery periods are likely to influence TDEE, yet longitudinal DLW data remain scarce. More studies that track athletes across multiple phases of the competitive year (e.g., including off-season, pre-season, and championship phases, etc.) are needed to better understand how energy demands evolve over time.

Despite these limitations, this review provides a rigorous synthesis of high-quality DLW data, offering valuable insights into sport-specific TDEE and PAL values in rugby and soccer athletes. Moreover, these findings highlight the sport-specific implications for nutritional planning and periodization to optimize performance and recovery strategies. Understanding sport-specific energy demands is essential for tailoring individualized nutritional recommendations.

## 5. Conclusions

TDEE is a dynamic and essential metric in athlete nutrition planning and training programming. Substantial variability in TDEE across sports and individuals underscores the need for personalized nutrition strategies. By incorporating PAL as a multiplier of RMR, practitioners can estimate TDEE in field settings. Aligning energy intake with individualized TDEE estimates is critical to optimizing athlete health, performance, and recovery. Continued research and practical application of TDEE and PAL will enhance the precision of sport nutrition support and help reduce the instances of energy deficiency in athletic populatio∗ns.

## Figures and Tables

**Table 1:** Subject characteristics of selected studies.

Study [Reference]	Sport	Sex	Subjects	Measurement Period	Height (cm) & Weight (kg)	Training/Match Details
Rugby						
Wilson et al., 2024 [31]	Rugby	Females	n = 15, Age (y) = 27 ± 2.6 Rugby Union players, Forwards & Backs	14 days (tournament)	Height: 170 ± 5.7 FFM: 59.6 ± 5.5 FM: 17.3 ± 6	14-day interval w/8 training days, three rest days, 1 travel day, 2 match days
Smith et al., 2018 [23]	Rugby	Males	n = 14 (5 U16, 5 U20, 4 U24), Age: U16 (15.2 ± 0.8), U20 (17.6 ± 1.1), U24 (23 ± 1.8) Rugby League Players	14 days (in-season)	Height: U16 (180.8 ± 7), U20 (176.8 ± 3.8), U24 (184.7 ± 2.5) Weight: U16 (79.3 ± 17.1), U20 (87.6 ± 8.8), U24 (98.3 ± 4.8)	2–9 light training days 1–7 heavy training days 0–2 match days 4–8 rest days
Smith et al., 2018 [23]	Rugby	Males	n = 13 [5 U16, 4 U20, 4 U24], Age: U16 (15.6 ± 0.5), U20 (18.3 ± 0.5), U24 (23 ± 0.8) Rugby Union Players	14 days (in-season)	Height: U16 (182.1 ± 7.5), U20 (178.1 ± 3.5), U24 (184.4 ± 3.2) Weight: U16 (85.4 ± 17.3), U20 (85.1 ± 8.3), U24 (99.4 ± 16.8)	0–3 light training days 3–7 heavy training days 0–2 match days 7–10 rest days
Smith et al., 2018 [23]	Rugby	Males	n = 10 [5 RL, 5 RU], Age: RL (15.2 ± 0.8), RU (15.6 ± 0.5) U16 Players (Combined Leagues)	14 days (in-season)	Height: RL (180.8 ± 7), RU (182.1 ± 7.5) Weight: RL (79.3 ± 17.1), RU (85.4 ± 17.3)	0–4 light training days 3–5 heavy training days 0–2 match days 8–10 rest days
Smith et al., 2018 [23]	Rugby	Males	n = 9 [5 RL, 4 RU], Age: RL (17.6 ± 1.1), RU (18.3 ± 0.5) U20 Players (Combined Leagues)	14 days (in-season)	Height: RL (176.8 ± 3.8), RU (178.1 ± 3.5) Weight: RL (87.6 ± 8.8), RU (85.1 ± 8.3)	1–2 light training days 4–7 heavy training days 0–2 match days 5–9 rest days
Smith et al., 2018 [23]	Rugby	Males	n = 8 [4 RL, 4 RU], Age: RL (23 ± 1.8), RU (23 ± 0.8) U24 Players (Combined Leagues)	14 days (in-season)	Height: RL (184.7 ± 2.5), RU (184.4 ± 3.2) Weight: RL (98.3 ± 4.8), RU (99.4 ± 16.8)	1–9 light training days 1–4 heavy training days 0–2 match days 4–9 rest days
Morehen et al., 2016 [22]	Rugby	Males	n = 6, Age: NA Rugby League Players, Forwards and Backs	14 days (in-season)	Height: 182.8 ± 2.7 Weight: 94.7 ± 6.7	2 weeks of structured training, including 4 rest days, 8 training days, 2 game days
Costello et al., 2022 [32]	Rugby	Males	n = 8 [6 pre-season, 7 in-season], Age: 17 ± 1 European Super League Academy	7 days (in-season) + 14 days (pre-season)	Height: 179.5 ± 8.7 Weight: 90.5 ± 11.4	Pre-season: 13 days with 10 training sessions, 10 field sessions, 4 rest days. In-season: 3 training sessions, 3 field sessions, 2 rest days, 1 match
Morehen et al., 2022 [24]	Soccer	Females	n = 24, Age: NA Professional International Players	12 days (pre-season)	Height: 168.1 ± 5.9 Weight: 62.1 ± 4.7	9-day training camp including 4 training days, 1 rest day, 2 travel days, 2 match days + 3 days at home
Dasa et al., 2023 [33]	Soccer	Females	n = 51, Age: 22 ± 4 Both professional and elite youth Norwegian players	14-day observational (in-season)	Height: 169 ± 7 Weight: 63.9 ± 6.6	1.7 ± 1.5 match days, and 10.7 ± 0.9 training days
Anderson et al., 2017 [25]	Soccer	Males	n = 6, Age:27 ± 3 Premier League	7 days (in-season)	Height: 180 ± 7 Weight: 80.5 ± 8.7	2 game days, 5 days “normal in-season training”
Hannon et al., 2021 [34]	Soccer	Males	n = 8, Age: 12.2 ± 0.4 U12/13 EPL Soccer Academy	14 days (in-season)	Height: 157.1 ± 4.1 Weight: 43.0 ± 4.8	6 rest days, 6 training days, 2 match days
Hannon et al., 2021 [34]	Soccer	Males	n = 8, Age: 15.0 ± 0.2 U15 EPL Soccer Academy	14 days (in-season)	Height: 173.9 ± 5.6 Weight: 56.8 ± 6.2	5 rest days, 6 training days, 3 match days
Hannon et al., 2021 [34]	Soccer	Males	n = 8, Age: 17.5 ± 0.4 U18 EPL Soccer Academy	14 days (in-season)	Height: 181.2 ± 5.2 Weight: 73.1 ± 8.1	4 rest days, 6 training days, 4 match days
Stables et al., 2023 [35]	Soccer	Males	n = 8, Age: 13.4 ± 0.2 Cat1 Premier League Academy	14 days (in-season)	Height: 165.7 ± 7.2 Weight: 51.2 ± 8.4	2 match days, 8 training days, 4 rest days
Stables et al., 2023 [35]	Soccer	Males	n = 6, Age: 13.1 ± 0.5 Non-Academy Players	14 days (in-season)	Height: 162.9 ± 6.4 Weight: 52.7 ± 12.4	2 match days, 2 training days, 10 rest days
Ebine et al., 2002 [21]	Soccer	Males	n = 7, Age: 22.1 ± 1.9 Professional Players	7 days (in-season)	Height: 175 ± 5 Weight: 69.8 ± 4.7	2 match days, 5 days “normal training regime”
Brinkmans et al., 2019 [26]	Soccer	Males	n = 41, Age: 23 ± 4 Dutch Eredivisie Pro Players (Total)	3–4 weeks (in-season)	Height: 182 ± 6 Weight: 77.6 ± 8.0	2.3 ± 0.5 matches played, 8.7 ± 1 training sessions, 3.1 ± 1 rest days over a 14-day study period
Brinkmans et al., 2019 [26]	Soccer	Males	n = 12, Age: 25 ± 4 Dutch Eredivisie (Defender)	3–4 weeks (in-season)	Height: 185 ± 4 Weight: 79.0 ± 7.4	
Brinkmans et al., 2019 [26]	Soccer	Males	n = 13, Age: 22 ± 4 Dutch Eredivisie (Midfielder)	3–4 weeks (in-season)	Height: 179 ± 5 Weight: 71.7 ± 4.9	
Brinkmans et al., 2019 [26]	Soccer	Males	n = 12, Age: 21 ± 3 Dutch Eredivisie (Attacker)	3–4 weeks (in-season)	Height: 181 ± 8 Weight: 78.5 ± 7.1	

FFM = fat-free mass; FM = fat mass; n = samplse size; RU = rugby union; RL = rugby league; EPL = English Premier League.

**Table 2:** Energetic data of individual studies analyzed.

Reference	Sport	N (Sex)	Skill	TDEE (kcal·kg^−1^·Day^−1^)	rTDEE (kcal/kg/Day)	RMR (kcal·d^−1^)	PAL
[31]	Rugby	15 (F)	Pro	3229 ± 545	NA	1578 ± 223	2.0 ± 0.3
[23]	Rugby	14 (M)	Pro	4369 ± 979	50 ± 10	2366 ± 296	1.90 ± 0.36
[23]	Rugby	13 (M)	Pro	4365 ± 1122	49 ± 9	2123 ± 269	2.07 ± 0.46
[23]	Rugby	10 (M)	U16	4010 ± 744	50 ± 8	2168 ± 353	1.91 ± 0.20
[23]	Rugby	9 (M)	U20	4414 ± 688	51 ± 9	2318 ± 335	1.93 ± 0.33
[23]	Rugby	8 (M)	U24	4761 ± 1523	48 ± 11	2232 ± 221	2.14 ± 0.64
[22]	Rugby	6 (M)	Pro	5378 ± 645	NA	1878 ± 96	2.86 ± 0.37
[32]	Rugby	7 (M)	Pro	3862 ± 184	NA	NA	NA
[32]	Rugby	6 (M)	Pro	4384 ± 726	NA	NA	NA
[24]	Soccer	24 (F)	Elite	2693 ± 432	43 ± 6	1504 ± 314	1.79 ± 0.24
[33]	Soccer	51 (F)	Pro	2918 ± 322	45.4	NA	2.00 ± 0.31
[25]	Soccer	6 (M)	Pro	3566 ± 585	NA	NA	NA
[34]	Soccer	8 (M)	U12	2859 ± 265	66.5 ± 9.6	1892 ± 211	1.5 ± 0.1
[34]	Soccer	8 (M)	U15	3029 ± 262	53.3 ± 7.4	2023 ± 162	1.5 ± 0.1
[34]	Soccer	8 (M)	U18	3586 ± 487	73.1 ± 8.1	2236 ± 93	1.6 ± 02
[35]	Soccer	8 (M)	Elite	3380 ± 517	66 ± 6	1824 ± 90	1.85 ± 0.30
[35]	Soccer	6 (M)	Youth	2641 ± 308	52 ± 10	1699 ± 45	1.55 ± 0.19
[21]	Soccer	7 (M)	Pro	3532 ± 408	50.6 ± 6.8	1674 ± 307	2.11 + 0.30
[26]	Soccer	41 (M)	Pro	3285 ± 354	42.4 ± 3.5	1877 ± 246	1.75 ± 0.13
[26]	Soccer	4 (M)	Pro	3365 ± 231	37.6 ± 2.9	2052 ± 215	1.64 ± 0.13
[26]	Soccer	12 (M)	Pro	3333 ± 489	42.0 ± 3.3	1894 ± 327	1.76 ± 0.16
[26]	Soccer	13 (M)	Pro	3180 ± 294	44.4 ± 3.2	1787 ± 204	1.78 ± 0.12
[26]	Soccer	12 (M)	Pro	3322 ± 297	42.4 ± 2.6	1888 ± 206	1.76 ± 0.11

NA = Not available; M = Males; F = Females; n = sample size; RMR = Resting metabolic rate; PAL = Physical activity level; rTDEE = Relative total daily energy expenditure; TDEE = Total daily energy expenditure; kg = Kilograms; kcal = kilocalories.

## Data Availability

No new data were created or analyzed in this study. Data sharing is not applicable to this article.

## Abbreviations

The following abbreviations are used in this manuscript:

(R)TDEE	(Relative) Total Daily Energy Expenditure
PAL	Physical Activity Level
RMR	Resting Metabolic Rate
PAEE	Physical Activity Energy Expenditure
NEAT	Non–Exercise Activity Thermogenesis
METs	Metabolic Equivalents
LEA	Low Energy Availability
RED–S	Relative Energy Deficiency in Sport
DLW	Doubly Labeled Water
